# Acute Stroke and Atrial Fibrillation: Risk of Incorrect NOAC Dosage When Estimating Renal Function From Plasma Creatinine Only

**DOI:** 10.3389/fneur.2022.907912

**Published:** 2022-07-05

**Authors:** Danial C. Amoey, Julia Thranitz, Thomas F. Münte, Georg Royl

**Affiliations:** ^1^Department of Neurology, University of Lübeck, Lübeck, Germany; ^2^Center of Brain, Behavior and Metabolism, University of Lübeck, Lübeck, Germany

**Keywords:** creatinine clearance, NOAC, stroke, atrial fibrillation, dosage adaptation, stroke unit, renal function

## Abstract

**Background:**

Cardioembolic stroke (CS) due to atrial fibrillation (AF) bears a high risk of unfavorable outcome. Treatment with a non-vitamin K antagonist oral anticoagulant (NOAC) reduces this risk. NOAC dosage occurs on a thin line during the acute phase of the stroke unit when the patient is threatened by both recurrent CS and a hemorrhagic stroke. It is often adapted to renal function—usually glomerular filtration rate (GFR)—to prevent both under- and overdosing. This study investigates the hypothetical risk of incorrect NOAC dosage after acute stroke when relying on plasma creatinine alone in comparison to a more exact renal function assessment including urine collection.

**Methods:**

In a cohort study on consecutive 481 patients treated in a stroke unit with acute stroke and AF, the GFR estimated from plasma creatinine (eGFR) was compared to concurrent creatinine clearance measurement (CrCl) from urine collection regarding the hypothetically derived NOAC dosage.

**Results:**

The risk of incorrect dosage (mean, 95% confidence interval) was 6.9% (4.8–9.5), 26% (23–31), 38% (33–42), and 20% (16–23) for apixaban, dabigatran, edoxaban, and rivaroxaban, respectively. The overall risk for incorrect dosage of any NOAC was 23% (21–25). Thresholds for age and admission eGFR were optimized to achieve an overall risk below 5% by additional CrCl measurements in selected patients (apixaban <36 ml/min and any age, dabigatran <75 ml/min and >70 y, edoxaban >36 ml/min and >58 y, rivaroxaban <76 ml/min and >75 y, any NOAC <81 ml/min and >54 y). The resulting portion of patients requiring an additional CrCl measurement was 10, 60, 80, 55, and 65% for apixaban, dabigatran, edoxaban, rivaroxaban, and any NOAC, respectively.

**Conclusions:**

There is a considerable risk of incorrect NOAC dosage in patients with acute CS treated in a stroke unit that can be lowered by targeted CrCl measurements in selected patients.

## Introduction

Cardioembolic stroke (CS) is of major concern because of its high risk of an unfavorable outcome (1, 2) which can be explained by the common pathomechanism of an embolic large-vessel occlusion occurring in an environment of weak collateral perfusion (3, 4). Patients with a CS have a higher risk of neurological deterioration in the prehospital phase (5). In addition, there is a high risk of recurrent stroke and symptomatic intracerebral hemorrhage in the early phase when patients usually are in a stroke unit which in turn often leads to uncertainty on when and how to start anticoagulant treatment (6), especially when considering the therapeutic challenge involved in intracerebral hemorrhage (7). For patients with atrial fibrillation (AF), the current guidelines advise preferring a non-vitamin K antagonist oral anticoagulant (NOAC) like apixaban, dabigatran, edoxaban, and rivaroxaban over a Vitamin K antagonist (VKA) due to the lower risk of both ischemic and hemorrhagic strokes (8). While this recommendation is derived from randomized trials involving long-term treatment, the situation in the acute stroke phase is unclear and subject to ongoing trials evaluating optimal therapy strategies (9).

While VKA dosage is usually driven by its measurable effect on coagulation, NOAC dosage is usually chosen after determining renal function. In the acute stroke phase, it is crucial to choose the correct NOAC and its dosage to find the “golden middle course” between the risk of ischemic stroke on the one side and the risk of hemorrhagic stroke on the other side, which has been shown to threaten patients with under- and overdosing of NOACs (10). Each NOAC has its thresholds for glomerular filtration rate (GFR) indicating the need for dose reduction or even contraindication (11). GFR can be measured by determining endogenous creatinine clearance (CrCl) but involves the collection of the urine which can be logistically demanding. For an approximation, different formulas for estimated GFR (eGFR) derive GFR from plasma creatinine concentrations alone. The primary objective of this study is to estimate the hypothetical risk of an incorrect NOAC dosage when relying on eGFR instead of CrCl.

## Methods

Clinical and laboratory data were collected by reviewing hospital records of consecutive stroke unit patients over 3.5-years (05/2014–12/2017) at University Hospital Schleswig-Holstein, Campus Lübeck (Lübeck, Germany). The study was performed as per the Declaration of Helsinki and after positive approval of the local ethical committee.

Inclusion criteria were an acute stroke (ischemic or hemorrhagic) or a transient ischemic attack with symptom onset up to 10 days before hospital admission and a history of or newly detected AF. Patients who were not able to collect urine, received hemodialysis or were in the process of dying with therapy limited to anxiolysis and analgesia were excluded from the study (41 patients). Altogether, 481 patients met the inclusion and exclusion criteria.

The local standard evaluation of stroke patients included a plasma creatinine measurement at admission. If patients had a history of or newly detected AF and were considered for treatment with NOAC, an additional plasma creatinine measurement at the end of a urine collection was done to assess GFR. This was done with two approaches. In one, eGFR was calculated from plasma creatinine alone using the CKD-EPI formula (12). The second approach measured CrCl from [Creatinine]_Urine_^*^ V_Urine_ / [Creatinine]_Plasma_. Both eGFR and CrCl were normalized to the body surface area calculated from the weight and height of the patient (13).

In addition to these laboratory data, the following parameters were collected: gender, height, body weight, relevant time points of stroke management (last seen well, admission, admission plasma creatinine measurement, CrCl measurement after admission), National Institutes of Health Stroke Scale (NIHSS) at admission, and stroke type.

To analyze the rate of incorrect NOAC dosage, eGFR and CrCl were evaluated within each patient to determine the resulting dosage of apixaban (reduced dosage at <30 ml/min, contraindication at <15 ml/min), dabigatran (reduced dosage at 30–50 ml/min, contraindication at <30 ml/min), edoxaban (reduced dosage at 30-−49 ml/min, contraindication at <30 ml/min and >100 ml/min), and rivaroxaban (reduced dosage at 15–49 ml/min, contraindication at <15 ml/min). The patient was categorized as “incorrect NOAC dosage” if the decision for or against a NOAC or the chosen dosage based on eGFR was different from the decision based on CrCl. For each NOAC, the absolute number of patients with incorrect dosage was divided by the absolute number of patients of the study cohort to obtain the risk of incorrect dosage. In addition, overall risk of incorrect NOAC dosage was estimated by pooling the data of all four NOACs. For each risk, a 95% confidence interval (CI) was added by the Clopper-Pearson method. To evaluate differences in eGFR from the CKD-EPI formula, eGFR was also calculated with three other common approaches: the MDRD formula (14), the BIS1 formula (15), and the Cockroft-Gault formula (16).

To identify possible risk factors for incorrect NOAC dosage, the obtained additional parameters mentioned above were tested for significant differences between the group with correct NOAC dosage and the group with incorrect NOAC dosage. Categorical variables were tested with Fisher's exact test (FET). Numerical variables were tested for normal distribution with a Shapiro–Wilk's test. If normally distributed, a pooled *t*-test (TT), if not normally distributed, a Wilcoxon's rank-sum test (WRS) was applied.

To determine a selected patient group in which additional CrCl measurements should be performed to most effectively decrease the risk of an incorrect NOAC dosage, an additional analysis was done. Since age and admission eGFR CKD-EPI were identified as risk factors (see Results section), we performed iterative calculation procedures using MATLAB^®^ (The MathWorks, USA). All reasonable threshold combinations for age and eGFR CKD-EPI were applied to obtain a 2D-matrix of the percentage of patients in which CrCl measurement would be performed with each matrix entry representing one threshold combination.

Along this matrix, isocurves of the same percentage (10–90% with 5% increases) were calculated. These isocurves were then applied to a second 2D-matrix analog to the percentage matrix which contained the resulting overall risk of incorrect NOAC dosage when performing an additional CrCl measurement in this subgroup. Within each isocurve, the point of lowest risk was identified. Following this, the threshold combination and percentage with additional CrCl measurement were obtained that decreased the risk to below 5%. From these data, a suggested algorithm was derived for each NOAC that gives thresholds for age and admission eGFR to select patients for additional CrCl measurements to decrease the risk of wrong NOAC dosage below 5%.

## Results

**Figure 1:**
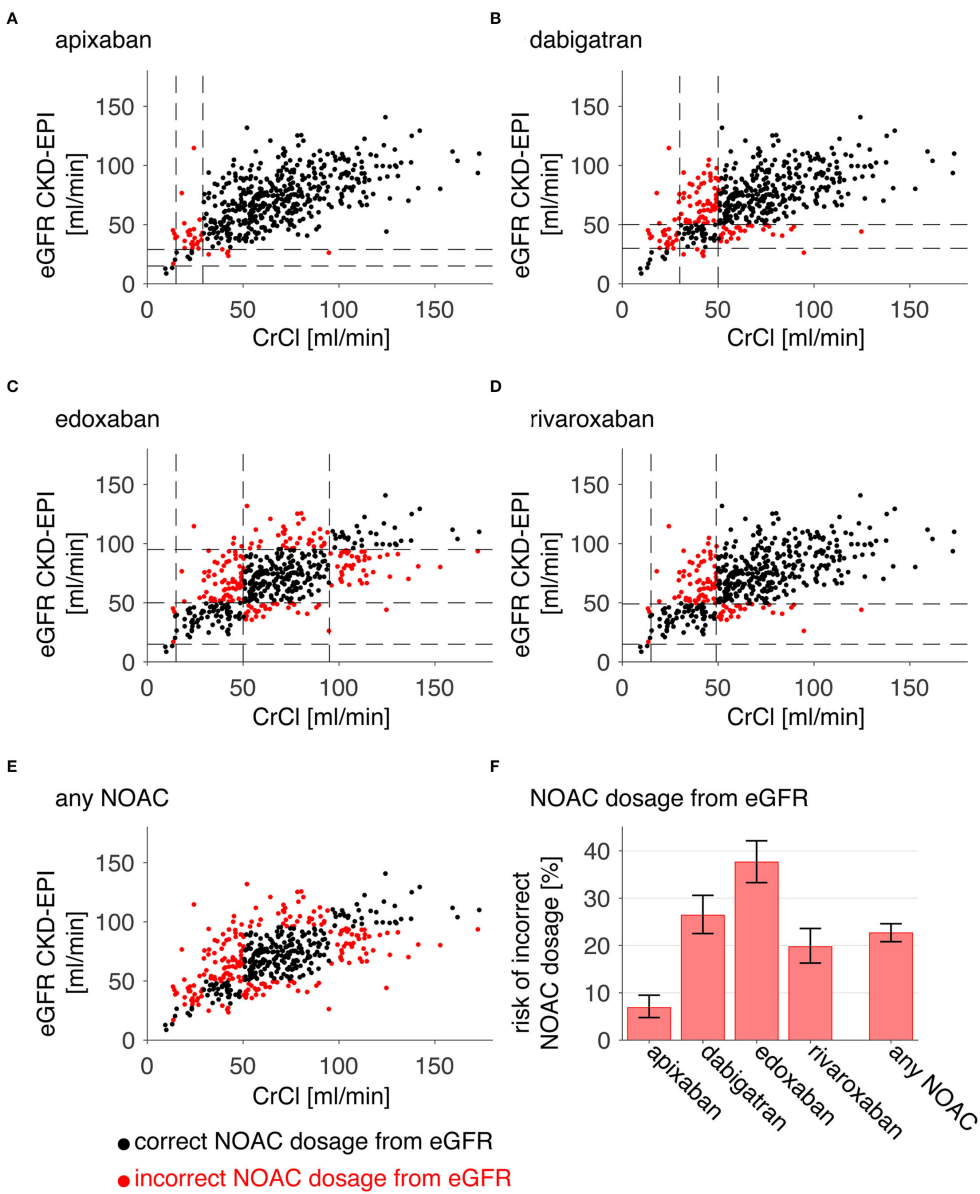
Risk of incorrect NOAC dosage. For each patient, eGFR CKD-EPI is plotted against CrCl **(A–E)**. Red points denote patients who would have an incorrect dosage or contraindication for apixaban **(A)**, dabigatran **(B)**, edoxaban **(C)**, rivaroxaban **(D)**, or either of these NOACs **(E)** when relying on eGFR CKD-EPI instead of CrCl [dashed lines in **(A–F)** mark the relevant GFR limits for the individual NOAC]. The relative portion of occurrences is plotted for each NOAC and all NOACS (“any NOAC”), and thus indicates the risk of an incorrect dosage (error bars are 95% confidence intervals derived from Clopper-Pearson intervals).

An overview of individual patient data comparing eGFR CKD-EPI with CrCl concerning NOAC dosage is displayed in Figure 1. Since different limits apply to each NOAC, a separate analysis was done for apixaban (a), edoxaban (b), dabigatran (c), and rivaroxaban (d). All patients in which a decision relying on eGFR CKD-EPI would differ from a decision based on CrCl (e.g., wrongful dose reduction or increase, non-observance of contraindication) are marked as red dots. There is a considerate number of patients with relevant deviations for each NOAC. Figure 1E shows deviation points across all NOACs. The risk of a wrong dosage (Figure 1F, mean and 95% CI) was 6.9% (4.8–9.5), 26% (23–31), 38% (33–42), and 20% (16–23) for apixaban, edoxaban, dabigatran, and rivaroxaban, respectively. When pooling the risks, a common risk for incorrect NOAC dosage of 23% (22–25) when relying on eGFR CKD-EPI was determined from the data. The data did not differ relevantly when applying eGFR MDRD, eGFR BIS1, or Cockroft-Gault formula instead of eGFR CKD-EPI (Table 1). Further analysis was done with eGFR CKD-EPI because of its numerically lowest risk for an incorrect dosage. The patient characteristics represent a typical Stroke Unit Cohort (Table 2).

**Table 1:** Comparison of different formulas to estimate glomerular filtration rate (eGFR) and resulting risks of incorrect NOAC dosage.

	**Risk of incorrect NOAC dosage with ±95% confidence interval**			
	**eGFR CKD-EPI**	**eGFR MDRD**	**eGFR BIS1**	**Cockcroft Gault**
Apixaban	6.9% 4.8–9.5%	7.1% 4.9–9.7%	7.1% 4.9–9.7%	8.1% 5.8–11%
Dabigatran	26% 23–31%	26% 22–30%	27% 23–31%	30% 25–34%
Edoxaban	38% 33–42%	41% 37–46%	37% 33–42%	40% 36–45%
Rivaroxaban	20% 16–24%	20% 17–24%	22% 18–26%	23% 20–27%
Any NOAC	23% 21–25%	24% 22–26%	23% 22–25%	25% 23–27%

*CKD-EPI, Chronic Kidney Disease Epidemiology Collaboration; MDRD, Modification of Diet in Renal Disease Study; BIS1, Berlin Initiative Study; Cockcroft Gault, Cockcroft Gault formula*.

**Table 2:** Variables of the 481 study patients and their distributions.

**Variable**	**Min**.	**Percentile**					**Max**.
		**2.5%**	**25%**	**50%**	**75%**	**97.5%**	
Age [y]	45	56	75	81	86	94	99
Height [cm]	140	151	164	170	178	188	195
Body weight [kg]	45	50	67	76	87	115	155
BSA [m^2^]	1.32	1.48	1.75	1.92	2.06	2.39	2.86
BMI (kg/m2)	17	20	24	26	29	39	51
Interval last seen well to adm. [hh:mm]	0:25	0:35	1:20	2:45	6:37	77:02	221:15
NIHSS at adm.	0	0	2	5	10	22	30
Interval adm. to adm. plasma creatinine meas. [hh:mm]	0:01	0:07	0:22	0:37	1:09	14:52	23:48
Adm. plasma creatinine [μmol/l]	33	52	74	89	108	181	525
Adm. eGFR CKD-EPI [ml/min]	9	24	50	66	84	116	143
Interval adm. to CrCl meas. [h]	13	24	43	64	105	234	400
Plasma creatinine at CrCl meas. [μmol/l]	33	48	69	85	103	179	450
GFR CKD-EPI at CrCl meas. [ml/min]	9	26	53	71	88	115	141

*min., minimum; max., maximum; BSA, body surface area; BMI, body mass index; adm., admission; meas., measurement*.

Table 3 compares variables of the subgroup with correct NOAC dosage to the subgroup with incorrect NOAC dosage when following eGFR CKD-EPI. While no significant differences are revealed for body size, weight, gender, and stroke type and severity, there are significant differences in several other variables. Patients with incorrect NOAC dosage derived from eGFR CKD-EPI were older than patients with correct NOAC dosage. They had a higher plasma creatinine and lower eGFR CKD-EPI at admission as well as at the time point of the CrCl measurement and the interval between hospital admission and CrCl measurement was larger in patients with incorrect NOAC dosage.

**Table 3:** Characteristics of study cohort and the subgroup of correct NOAC dosage when relying on eGFR as opposed to the subgroup of incorrect NOAC dosage when relying on eGFR.

	**Variable**	**Perc. / mean / median**	**All patients (*n* = 481)**	**Correct NOAC dosage with eGFR (*n* = 372)**	**Incorrect NOAC dosage with eGFR (*n* = 109)**	**Statistics**	
						**Test**	***p*-value**
Physical data	Male sex	Perc.	43%	41%	47%	FET	1
	Age [y]	Median	81	80	82	WRS	0.0001
	Height [cm]	Median	170	170	170	WRS	1
	Body weight [kg]	Median	76	76	79	WRS	1
	BSA [m^2^]	Mean	1.91	1.91	1.92	TT	1
	BMI (kg/m2)	Median	26	26	26	WRS	1
Stroke type and severity	Interval last seen well to adm. [hh:mm]	Median	2:45	2:45	3:00	WRS	1
	NIHSS at adm.	Median	5	5	4	WRS	1
	TIA	Perc.	12%	12%	13%	FET	1
	Ischemic infarction	Perc.	85%	85%	85%	FET	1
	Intracerebral hemorrhage	Perc.	2%	2%	2%	FET	1
Lab. analysis	Interval adm. to adm. plasma creatinine meas. [hh:mm]	Median	0:37	0:36	0:40	WRS	0.015
	Adm. plasma creatinine [μmol/l]	Median	89	87	96	WRS	0.00000012
	Adm. eGFR CKD-EPI [ml/min]	Mean	67	69	61	TT	0.000000020
	Interval adm. to CrCl. meas. [h]	Median	64	63	68	WRS	0.58
	Plasma creatinine at CrCl meas. [μmol/l]	Median	85	83	92	WRS	0.00000000016
	GFR CKD-EPI at CrCl meas. [ml/min]	Mean	71	73	64	TT	0.00000000018
	Pat. with 12 h instead of 24 h urine collection	Perc.	28%	28%	27%	FET	1

*lab., laboratory; perc., percentage; BSA, body surface area; BMI, body mass index; adm., admission; meas., measurement; FET, Fisher's exact test; WRS, Wilcoxon rank-sum test; TT, pooled t-test*.

**Figure 2:**
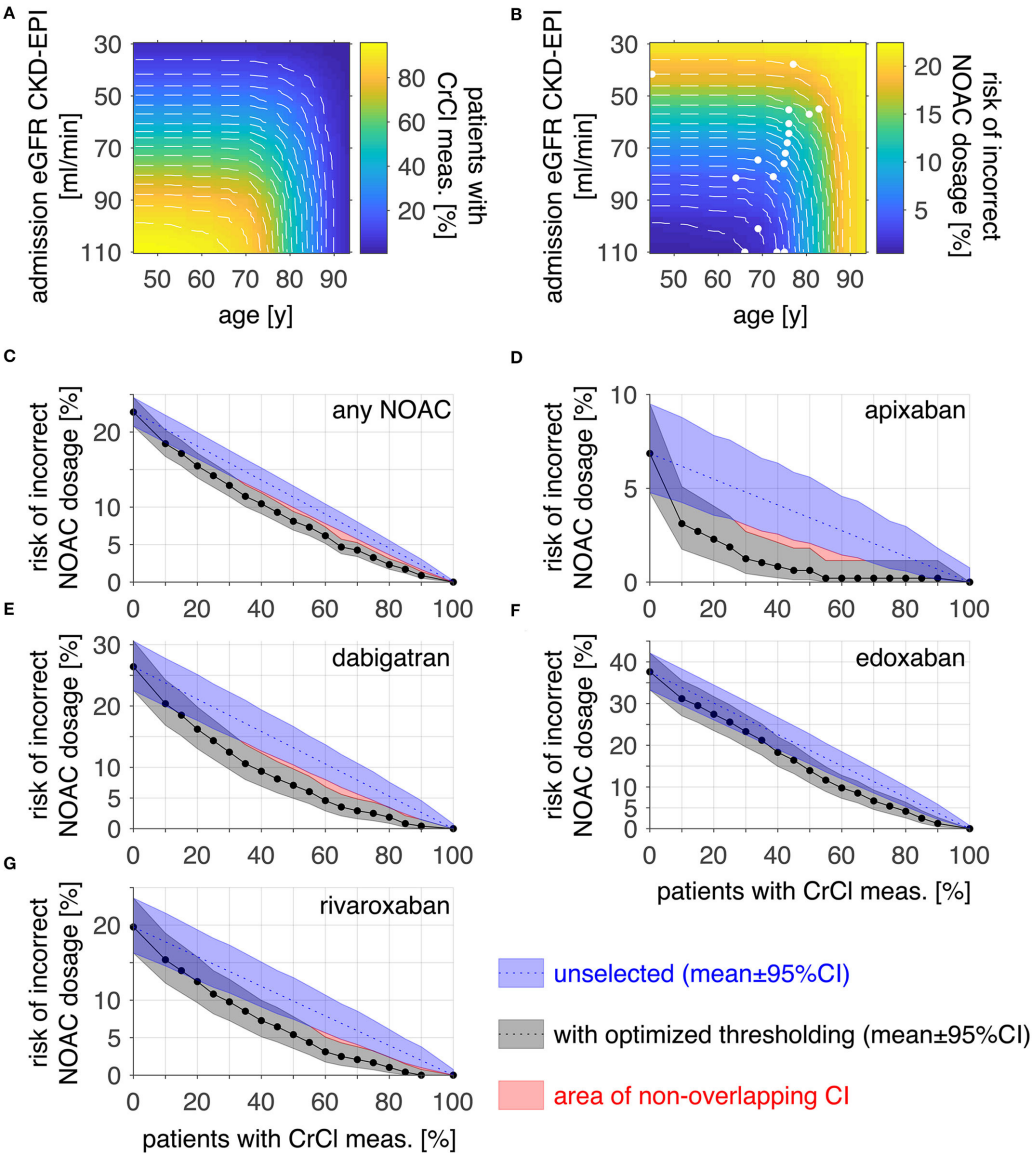
Optimizing thresholds for risk reduction by CrCl measurement. The proportion **(A)** and risk of incorrect NOAC dosage [**(B)**, any NOAC] of the patients above an admission eGFR CKD-EPI and below an age threshold are displayed as color-coded images. Threshold combinations of equal proportions [white dashed plots in **(A)**] were evaluated for the lowest risk of incorrect dosage [white points in **(B)**] to find the optimal thresholds for risk reduction. **(C)** displays the risk of incorrect NOAC dosage within the patient population when performing CrCl measurements across a portion of the population for an unselected (blue) group and a selection fulfilling the threshold criteria in **(B)** (gray). To reduce the risk of incorrect NOAC dosage in the whole population below 5%, the number of patients with additional CrCl measurement would have to be 65% after thresholding and 80% for unselected patients for any NOAC. **(D–G)** shows the corresponding analysis for apixaban (10 vs. 30%), dabigatran (60 vs. 85%), edoxaban (80 vs. 90%), and rivaroxaban (55 vs. 75%). Risk data are plotted as mean ± 95% CI, red areas denote non-overlapping CI; meas., measurement.

Since low eGFR CKD-EPI and high age were identified as predictors for an incorrect NOAC dosage, thresholds for these variables were evaluated to find a subgroup of patients in which an additional CrCl measurement would be most effective to reduce the risk of incorrect NOAC dosage (Figure 2). After selection of patients from admission eGFR CKD-EPI below increasing thresholds (y-axis) and with an age above decreasing thresholds (x-axis), Figure 2A color-codes the resulting percentage of patients with additional CrCl measurement whereas Figure 2B color-codes the resulting overall risk of incorrect NOAC dosage in the patient population.

Threshold combinations resulting in equal proportions were identified and are visible within the images as white dashed lines. Along these, the threshold combination with the lowest resulting risk was identified (white points in b). In Figure 2C, the risk of incorrect NOAC dosage for any NOAC in the patient cohort is plotted against the corresponding percentage of patients with additional CrCl measurement for selected patients with optimized thresholds (gray) as determined in 2b. For comparison, the risk when doing the additional CrCl measurement in an unselected patient group is plotted additionally (blue). For a risk reduction below 5% for any NOAC, the proportion of patients for which an additional CrCl measurement would be required is 65% for optimized thresholds compared to 80% in the unselected patient group. The corresponding analysis was done for each NOAC separately. The results are displayed in Figures 2D–G (image data corresponding to a and b not shown). The corresponding required proportion of patients for a risk below 5% was 10 vs. 30% for apixaban (d), 60 vs. 90% for dabigatran (e), 80 vs. 90% for edoxaban (f), and 55 vs. 75% for rivaroxaban (g).

**Figure 3:**
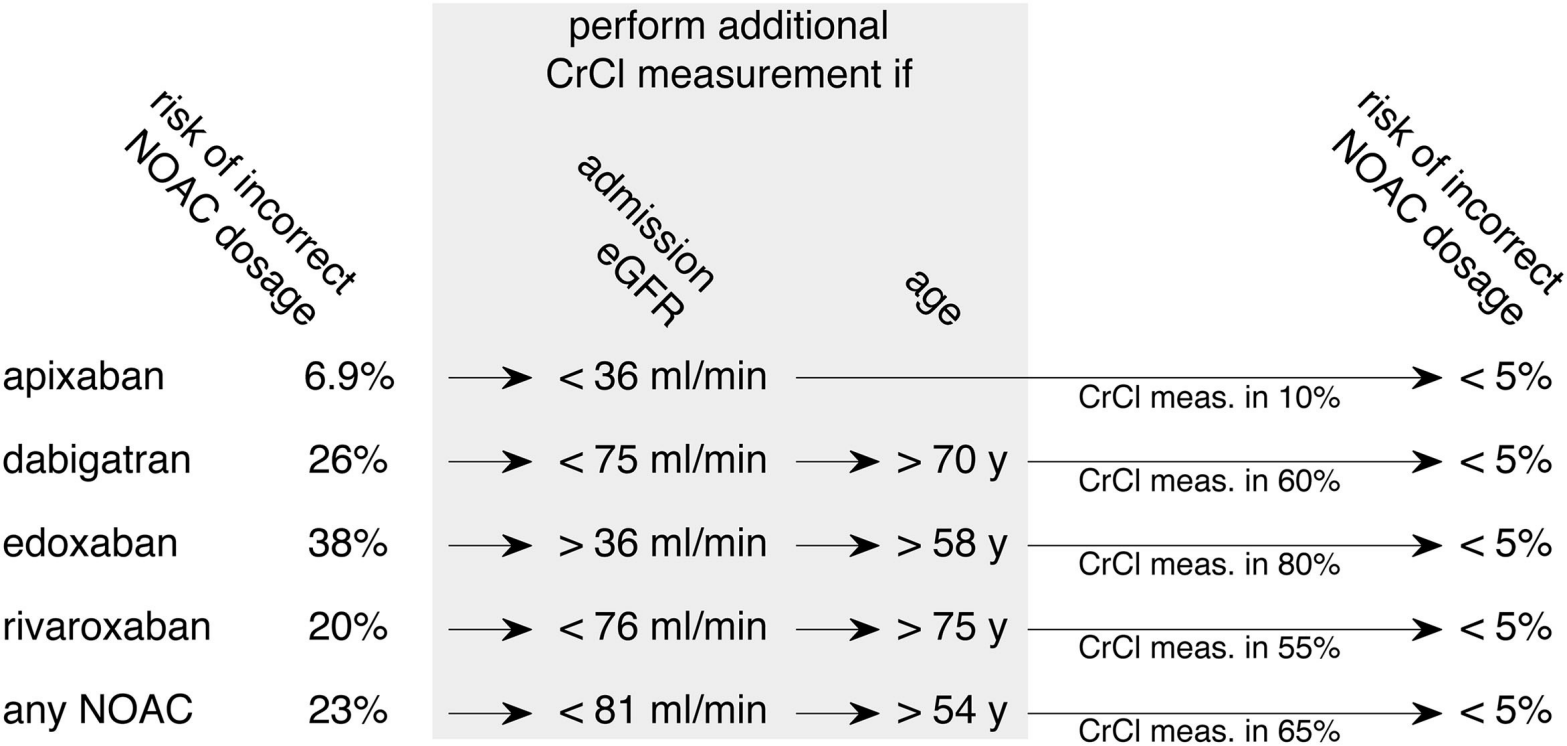
Derived clinical pathway to decrease the risk of incorrect NOAC dosage. Aiming at a reduction of incorrect NOAC dosage below 5%, a clinical decision rule for additional CrCl measurement based on thresholds of the data is displayed. The optimal selection of patients can be adjusted to the individual NOAC that is chosen for therapy (upper four rows). An alternative approach is to consider all NOACs equally when deciding which patients to select for additional CrCl measurement (bottom row). Admission eGFR contains results from eGFR CKD-EPI; meas., measurement.

Based on this thresholding analysis, a clinical decision rule is suggested as displayed in Figure 3. When considering a specific NOAC, the selection criteria for an additional CrCl measurement are given to achieve a risk of incorrect dosage below 5%. For apixaban, which has the lowest risk for incorrect dosage (6.9%), it is sufficient to perform an additional CrCl measurement in patients of any age with an eGFR CKD-EPI on the admission of <36 ml/min (representing 10% of patients) to reduce the risk of incorrect dosage to <5%. The corresponding thresholds for dabigatran (26% risk of incorrect dosage without CrCl measurement) are an admission eGFR CKD-EPI <75 ml/min and patient age >70 y. In this case, the selected group included 60% of all patients. The overall risk of incorrect dosage for edoxaban (38%) can be reduced to below 5% by acquiring CrCl in patients with an admission eGFR CKD-EPI >36 ml/min and age > 58 y which applied for 80% of all patients. When treating with rivaroxaban, the risk of incorrect NOAC dosage (20%) can be reduced to <5% by measuring CrCl in patients with an admission eGFR CKD-EPI <76 ml/min and age >75 y, amounting to 55% of all patients. If the selection of patients for CrCl measurement is done before selecting a specific NOAC, the overall risk of incorrect dosage of 23% can be reduced to <5% by including patients with an admission eGFR CKD-EPI <81 ml/min and age >54 y, which is equivalent to 65% of the study cohort.

## Discussion

In a cohort study on 481 stroke unit patients with AF, we have estimated a risk of 23% for an incorrect NOAC dosage when relying on eGFR instead of CrCl. The risk varies with the type of NOAC (apixaban 6.9%, dabigatran 26%, edoxaban 38%, and rivaroxaban 20%). In addition, it correlates positively with age and negatively with admission eGFR. Performing an additional CrCl measurement in a portion of patients selected by age, admission eGFR, and type of NOAC considered for therapy, can reduce the risk of incorrect NOAC dosage below 5%. In this study, the portion was low for apixaban (10%, patients of any age with admission eGFR <36 ml/min), intermediate for dabigatran (60%, patients with admission eGFR <75 ml/min and age <70 y), rivaroxaban (55%, patients with admission eGFR <76 ml/min and age >75 y), and comparatively high for edoxaban (80%, all patients with admission eGFR >36 ml/min and age >58 y). It should be noted that the recommendation to not apply edoxaban in a patient with GFR >100 ml/min is stated in the 2018 EHRA guidelines. Following the latter guideline, GFR thresholds for dose reduction or contraindication would be equal to rivaroxaban. Therefore, when abolishing the upper GFR limit of 100 ml/min, the risk of an incorrect edoxaban dosage would be 20%. When selecting patients with an eGFR <76 ml/min and an age >70 y, this would cause a portion of 55% with an additional CrCl measurement necessary to decrease the risk of an incorrect dosage below 5%.

Our study has several limitations. It analyzes a local standard of care at our stroke unit with a standard follow-up assessment of renal function. While admission eGFR was done between 1 min and 24 h after admission with a median of 37 min, the time point for follow-up eGFR varied considerably, between 13 h and 17 d (median 64 h). The reason for this variance is that follow-up eGFR was only done if AF was known or diagnosed for the first time and NOAC treatment was therefore considered. The mean eGFR at admission was 67 ml/min while mean eGFR at follow-up was 71 ml/min. Considering this, our suggested algorithm requires a follow-up eGFR in any case, even if no CrCl measurement is involved. The optimal time point for this follow-up eGFR cannot be determined from our data, but the “real-world” management of performing eGFR follow-up as soon as NOAC treatment is considered may be a reasonable approach.

A further limitation is that our risk analysis was done in a local population of stroke patients. Although it is a non-selected patient cohort from a standard tertiary hospital stroke unit, the derived selection thresholds for CrCl measurements may be slightly different in other stroke care centers depending on, e.g., population age and prevalence of renal diseases in the average patient population. However, it is improbable that the overall risk of incorrect NOAC dosage differs relevantly. While it may be feasible for some stroke centers to perform CrCl measurements in all patients with NOAC treatment routinely (with maximal risk reduction), this may not be a practical procedure for all stroke centers. Our suggested algorithm helps to select patients that benefit most from CrCl measurement.

In our study, CrCl measurement was considered a gold standard. This has been done for other studies as well. For instance, when dosing vancomycin in critically ill patients, CrCl has been shown to outperform eGFR (17). While, compared to eGFR approaches, CrCl has the advantage of adding urine creatinine as a second parameter, its accuracy can be impairedby both methodological errors like tubular secretion of creatinine (18) and execution errors (e.g., collection time and logistics). An alternative approach that could be followed is the measurement of Cystatin C, an endogenous marker that is completely filtrated in the glomeruli (18). This method is generally expensive and insufficiently standardized but could be a valuable addition in patients in which CrCl results are questioned, e.g., because of a high amount of muscle tissue (19).

There have been several approaches to compare different eGFR formulas in patients for NOAC dosage findings. Manzano-Ferndandez et al. compared different eGFR formulas (20) and found more discordance in the resulting dabigatran and rivaroxaban dosages than in the resulting apixaban dosages. Cemin et al. defined eGFR CKD-EPI as the gold standard and found that when adjusting NOAC dosage based on the Cockroft-Gault formula in comparison there was a relevant renal function class misclassification (21). If the Cockroft-Gault formula is considered the gold standard, as it was done in a study by Simpson et al. (22), there is an overestimation of renal function when based on eGFR CKD-EPI instead in the elderly population. The question of whether eGFR CKD-EPI or the Cockroft-Gault formula should be considered as the gold standard cannot be answered by these studies. The Cockroft-Gault formula has been employed in some but not all the relevant clinical NOAC trials (23–26) to assess renal function. However, while it is one of the first approaches to eGFR estimation, its validity and reliability has been challenged with evidence for a better outcome when directing dosage with eGFR CKD-EPI and MDRD (27). While our study also suggests a trend toward inferiority of the Cockroft-Gault formula to eGFR CKD-EPI, it follows a different approach by using CrCl with parallel urine creatinine measurement as the gold standard. Therefore, it looks at the general weakness of renal function assessment based on plasma creatinine alone when comparing it to a more extensive laboratory estimate CrCl measurement involving parallel urine creatinine measurement.

Our study investigated the risk of incorrect NOAC dosage in stroke patients in the acute phase, a period when both ischemic and hemorrhagic events are frequent and a major target of prevention. When managing patients with AF, the treating physician has to consider that NOAC therapy decreases the risk of an ischemic stroke and increases the risk of hemorrhagic events. Overall, the superiority of NOACs over VKAs in risk/benefit ratios appears robust even in patients with presumed incorrect NOAC dosage (28, 29), although a relevant portion of this effect may be an insufficient time in the therapeutic range in patients treated with VKA (30). Recent data from the ORBIT AF trial showed that there is a risk for NOAC overdosage resulting in increased rates of bleeding events in patients with a decline in renal function over time (31). Looking from the side of embolic risk, the PAVE-AF antithrombotic study revealed a high portion of NOAC underdosage (39% apixaban, 7% dabigatran, and 26% rivaroxaban) in the elderly population (32). The expected effect of a higher rate of ischemic strokes when apixaban is underdosed is suggested by a recent large Danish register study (33). For these reasons, we weighted under- and overdosage to the same degree in our study. From a safety point of view, it appears reasonable to favor apixaban in acute stroke patients due to the low *a priori* risk of incorrect dosage (6.7%). In line with this, a recent study on Medicare data reported decreased risks of stroke and major bleeding in patients with chronic kidney disease when treated with apixaban compared to warfarin, rivaroxaban, and dabigatran (34).

In our study, we applied NOAC dosing following GFR limits as recommended by EHRA guidelines (35). While doses for apixaban, edoxaban, and rivaroxaban were adjusted to renal function in their respective randomized trials (ROCKET-AF, ARISTOTLE, and ENGAGE-AF), dabigatran doses were randomly assigned in the corresponding trial regardless of the renal function (RE-LY) with subgroup analysis eventually leading to the recommended summary of product characteristics (23–26). Considering this, it is a legitimate question whether incorrect dosage following renal function is in fact an incorrect dosage. Furthermore, the hepatic function is also relevant, as all NOACs are contraindicated in significant hepatic insufficiency like Child-Turcotte-Pugh C cirrhosis (11).

For apixaban, EHRA guidelines state that—along with the ARISTOTLE and AVERROES trials—an alternative and more simple approach is valid for dosage finding (11, 23, 36). Following this approach, apixaban dosage can be reduced to 2 × 2.5 mg if two of the following criteria apply: age ≥ 80 years, body weight ≤ 60 kg, serum creatinine ≥ 133 μmol/l. To investigate the effect of this algorithm, we performed an additional analysis in which we screened our sample of 481 patients and removed all patients fulfilling two of the three criteria (age ≥ 80 years, body weight ≤ 60 kg, creatinine ≥ 133 μmol/l) from the database, assuming that the decision would be made without additional renal function assessment. From our 481 patients, 87 patients fulfilled these criteria. When restricting the analysis in Figure 1A for apixaban to the remaining 394 patients, the mean risk of an incorrect dosage (95% confidence interval) was reduced from 6.9% (4.8–9.5) to 4.1% (2.4–6.5). Therefore, it may be argued that in patients in which apixaban is considered, dose reduction should be done in patients fulfilling two of the three criteria (age ≥ 80 years, body weight ≤ 60 kg, creatinine ≥ 133 μmol/l). In all other patients, dose direction based on eGFR would be accurate enough to reduce the risk of an incorrect dosage below 5%. However, there are also arguments in favor of using renal function measurement for all patients. For one, apixaban approval studies were done on patients in a steady state long-term environment where the applied clinical criteria might sufficiently approximate the overall renal function (23, 36). Patients with an acute stroke differ from this group since the renal function itself often becomes unstable, e.g., due to exsiccosis, heart failure, or other causes. Another point to consider is the fact that while applying clinical criteria may be used to identify patients requiring the lower 2 × 2.5 mg dosage of apixaban, it does not identify patients with a renal function impairment with a CrCl below 15 ml/min in whom apixaban treatment is contraindicated. Taken together, we see convincing arguments to prefer renal function measurement to clinical criteria in patients in the immediate phase after an acute stroke when directing apixaban treatment and dosage. The main result of our study is in fact that apixaban is a relatively safe NOAC candidate when estimating eGFR based on plasma creatinine. Only in the subset of patients (10% in our cohort) with an eGFR <36 ml/min, an additional CrCl measurement should be done to decrease the risk of an incorrect dosage below 5%.

In principle, NOAC serum concentrations could be used instead of renal function estimates to guide NOAC dosage. However, the analysis is not widely and readily available and there is no standard definition of what NOAC serum concentration should be aimed at (37). Another approach would be to use coagulation assays like ecarin clotting or diluted thrombin time for dabigatran or calibrated anti-Xa testing for apixaban, edoxaban, and rivaroxaban (38). However, due to a lack of reliable reference ranges, they are not commonly used for NOAC dose adjustment, but rather for qualitative assessments, e.g., when planning a surgical emergency procedure or before thrombolysis, wherein the case of rivaroxaban, even point-of-care testing is a reasonable approach (39). Thus, to prevent off-label use and over- as well as underdosage of NOACs, the assessment of renal function remains the main pillar to date.

## Conclusion

For an optimal treatment of patients with a risk of CS due to AF, a correct NOAC dosage is mandatory to minimize the likelihood of both ischemic and hemorrhagic events, especially in the acute phase. When selecting NOAC and dosage, relying on eGFR bears a considerable risk of a wrongful decision that can be substantially reduced by measuring CrCl, either in all patients or in a selected group with a higher risk for incorrect dosage.

## Data Availability Statement

The datasets used and/or analyzed during the current study are available from the corresponding author on reasonable request.

## Ethics Statement

The study was performed in accordance with the Declaration of Helsinki and after positive approval of the local Ethical Committee, Ethikkommission der Universität zu Lübeck, Lübeck, Germany. As it relies on anonymized data from clinical routine, a written informed consent was not required.

## Author Contributions

DA and GR contributed to the study design and drafted the initial version. GR and JT did the statistical analysis. All authors were involved in data collection, interpretation and drafting of the manuscript, critically reviewed the manuscript, and approved the final draft.

## Funding

GR was funded by the European Union (Interreg5a). TM was funded by the DFG (MU1311/20-1, 17-1).

## Conflict of Interest

GR received speaker's honoraria and reimbursement for congress traveling and accommodation from Boehringer-Ingelheim, Bristol-Myers Squibb, and Daiichi Sankyo. The remaining authors declare that the research was conducted in the absence of any commercial or financial relationships that could be construed as a potential conflict of interest.

## Publisher's Note

All claims expressed in this article are solely those of the authors and do not necessarily represent those of their affiliated organizations, or those of the publisher, the editors and the reviewers. Any product that may be evaluated in this article, or claim that may be made by its manufacturer, is not guaranteed or endorsed by the publisher.

## Abbreviations

AF: Atrial Fibrillation
CI: Confidence Interval
Cockroft-Gault: Cockcroft Gault formula
CrCl: Creatinine Clearance
eGFR: estimated glomerular filtration rate
eGFR BIS1: eGFR from BIS1 formula
eGFR CKD-EPI: eGFR from CKD-EPI formula
eGFR MDRD: eGFR from MDRD formula
FET: Fisher's exact test
GFR: glomerular filtration rate
NIHSS: National Institutes of Health Stroke Scale
NOAC: non-vitamin K antagonist oral anticoagulant
TT: pooled t-test
VKA: Vitamin K antagonist
WRS: Wilcoxon rank-sum test.
